# Dynamic cerebral autoregulation during step-wise increases in blood pressure during anaesthesia: A nonrandomised interventional trial

**DOI:** 10.1097/EJA.0000000000001798

**Published:** 2023-02-06

**Authors:** Rokus E.C. van den Dool, Nicolaas H. Sperna Weiland, Jimmy Schenk, Eline Kho, Denise P. Veelo, Björn J.P. van der Ster, Rogier V. Immink

**Affiliations:** From the Department of Anaesthesiology, Amsterdam UMC, University of Amsterdam, Amsterdam, The Netherlands (RECD, NHSW, JS, EK, DPV, BJPS, RVI)

## Abstract

**BACKGROUND:**

Classically, cerebral autoregulation (CA) entails cerebral blood flow (CBF) remaining constant by cerebrovascular tone adapting to fluctuations in mean arterial pressure (MAP) between ∼60 and ∼150 mmHg. However, this is not an on–off mechanism; previous work has suggested that vasomotor tone is proportionally related to CA function. During propofol-based anaesthesia, there is cerebrovascular vasoconstriction, and static CA remains intact. Sevoflurane-based anaesthesia induces cerebral vasodilation and attenuates CA dose-dependently. It is unclear how this translates to dynamic CA across a range of blood pressures in the autoregulatory range.

**OBJECTIVE:**

The aim of this study was to quantify the effect of step-wise increases in MAP between 60 and 100 mmHg, using phenylephrine, on dynamic CA during propofol- and sevoflurane-based anaesthesia.

**DESIGN:**

A nonrandomised interventional trial.

**SETTING:**

Single centre enrolment started on 11 January 2019 and ended on 23 September 2019.

**PATIENTS:**

We studied American Society of Anesthesiologists (ASA) I/II patients undergoing noncardiothoracic, nonneurosurgical and nonlaparoscopic surgery under general anaesthesia.

**INTERVENTION:**

In this study, cerebrovascular tone was manipulated in the autoregulatory range by increasing MAP step-wise using phenylephrine in patients receiving either propofol- or sevoflurane-based anaesthesia. MAP and mean middle cerebral artery blood velocity (MCA*V*_mean_) were measured in ASA I and II patients, anaesthetised with either propofol (*n* = 26) or sevoflurane (*n* = 28), during 10 mmHg step-wise increments of MAP between 60 and 100 mmHg. Static CA was determined by plotting 2-min averaged MCA*V*_mean_ versus MAP. Dynamic CA was determined using transfer function analysis and expressed as the phase lead (°) between MAP and MCA*V*_mean_ oscillations, created with positive pressure ventilation with a frequency of 6 min^−1^.

**MAIN OUTCOMES:**

The primary outcome of this study was the response of dynamic CA during step-wise increases in MAP during propofol- and sevoflurane-based anaesthesia.

**RESULTS:**

MAP levels achieved per step-wise increments were comparable between anaesthesia regiment (63 ± 3, 72 ± 2, 80 ± 2, 90 ± 2, 100 ± 3 mmHg, and 61 ± 4, 71 ± 2, 80 ± 2, 89 ± 2, 98 ± 4 mmHg for propofol and sevoflurane, respectively). MCA*V*_mean_ increased more during step-wise MAP increments for sevoflurane compared to propofol (*P*≤0.001). Dynamic CA improved during propofol (0.73° mmHg^−1^, 95% CI 0.51 to 0.95; *P* ≤ 0.001)) and less pronounced during sevoflurane-based anaesthesia (0.21° mmHg^−1^ (95% CI 0.01 to 0.42, *P* = 0.04).

**CONCLUSIONS:**

During general anaesthesia, dynamic CA is dependent on MAP, also within the autoregulatory range. This phenomenon was more pronounced during propofol anaesthesia than during sevoflurane.

**TRIAL REGISTRATION:**

NCT03816072 (https://clinicaltrials.gov/ct2/show/NCT03816072).

**KEY POINTS**

- Cerebral autoregulation (CA) maintains cerebral blood flow more or less constantly across a wide range of mean arterial pressures.
- Cerebrovascular tone may be proportionally related to CA efficacy, as shown by the effects of vasoactive events (e.g. hypo- or hypercapnia) on CA. Specifically, vasodilation seems to impair dynamic CA whereas vasoconstriction appears to improve it.
- We manipulated cerebrovascular tone by step-wise increases in mean blood pressure using phenylephrine during either propofol- or sevoflurane anaesthesia, and studied its effect on CA.
- We found that increasing blood pressure improved CA in both propofol- and sevoflurane-anaesthesia, but this was more pronounced with propofol (a cerebral vasoconstrictor) compared with sevoflurane (a vasodilator).

## Introduction

An intra-operative mean arterial blood pressure (MAP) below 65 mmHg has been associated with organ hypoperfusion and postoperative complications.^1^ For the brain, this finding is consistent with the dogmatic physiological concept that cerebral (pressure) autoregulation (CA) maintains constancy of cerebral blood flow (CBF), by adjusting cerebrovascular tone to changes in MAP down to a lower limit (LLCA) of approximately 60 mmHg.^2^ In reality, this idea of ‘static’ CA is much more nuanced but remains a widespread model that serves as a basis for clinicians to make clinical decisions.^3^ Conversely, dynamic CA describes how quickly cerebrovascular tone is adjusted to a sudden change in MAP.^4^

Factors that influence cerebrovascular tone may interfere with CA.^5^ For example, arterial carbon dioxide tension is inversely related to cerebrovascular tone and hypercapnia has been found to attenuate CA, whereas hypocapnia improves it.^6,7^ Intra-operatively, high-dose isoflurane reduces cerebrovascular tone and impairs CA, whereas propofol-based anaesthesia increases cerebrovascular tone and maintains CA independently of the dose.^8,9^ Moreover, impaired CA during volatile anaesthesia can be improved by hypocapnia.^10^ It seems therefore that CA efficacy is positively related to cerebrovascular tone. Nevertheless, neurovascular coupling seems to be preserved during both propofol and volatile anaesthesia,^11,12^ although CBF is relatively increased compared to the metabolic rate of oxygen (CMRO_2_) during volatile anaesthesia.^13^

Another way to increase cerebrovascular tone is to increase MAP within the autoregulatory range, as famously demonstrated by Fog in several experiments on animals in the 1930s,^14–17^ later confirmed in humans with noninvasive methods.^18^ We hypothesised that dynamic CA is dependent on cerebrovascular tone even in the autoregulatory range. The aim of this study was to quantify the effect of step-wise increases in MAP between 60 and 100 mmHg using phenylephrine on dynamic CA during propofol- and sevoflurane-based anaesthesia.

## Methods

### Ethics

The Institutional Ethics Committee of the Academic Medical Centre AMC Amsterdam (Meibergdreef 9, 1105 AZ, Amsterdam) approved the study on 7 January 2019 (reference: MEC 2018_230), and the study was registered with ClinicalTrials.gov (ref: NCT03071432). Written informed consent was obtained from every patient prior to inclusion.

### Subjects

Patients with American Society of Anesthesiologists (ASA)-I or ASA-II classification, scheduled for elective, noncardiothoracic, nonneurosurgical and nonlaparoscopic surgery under general anaesthesia were eligible for inclusion in this prospective, nonrandomised interventional trial. Patients at high risk of autonomic dysfunction (e.g. diabetes, uncontrolled hypertension, Parkinson's disease), cardiac arrhythmias, and known cardiomyopathy were excluded.

### Instrumentation

Beat-to-beat arterial MAP was obtained using a servo-controlled finger photoplethysmograph (^cc^Nexfin, Edwards Lifesciences, Irvine, CA, USA) with the cuff placed at the mid-phalanx of the middle finger of either hand. Validation studies have shown the reliability of this noninvasive device to measure blood pressure during cardiac^19^ and vascular^20^ surgery across a wide range of blood pressures. Heart rate (HR) was calculated as the inverse of the beat-to-beat interval. In addition, stroke volume (SV) was estimated by cardiac output (CO)-trek, an algorithm that calculates SV by dividing the systolic area under the arterial curve by estimated aortic impedance.^21^ CO was computed by multiplying SV by HR. Cerebrovascular resistance index (CVRi) was calculated by dividing MAP by mean middle cerebral artery blood velocity.

The middle cerebral artery (MCA) was insonated unilaterally through the temporal window using a 2 MHz probe (Compumedics DWL Germany GmbH, Singen, Germany) attached to a headband (DWL Diamon Probe Fixation System; Compumedics DWL Germany GmbH). Insonation depth and signal gain were optimised manually. In order to find the MCA we followed standard procedures as described by Aaslid *et al.*^22^ In short, we placed the Doppler probe on the temporal region of the cranium and explored the transcranial window manually. First, the zygomatic arch was palpated to find the most caudal boundary of the temporal window. The ventral boundary was set as the anterior process of the zygomatic bone. The dorsal boundary was set as the pre-auricular area. The cranial boundary was approximately 3 cm from the zygomatic arch. The goal was to identify the most proximal segment of the MCA just as it leaves the circle of Willis. Normally, the bifurcation of the MCA and the anterior cerebral artery (ACA) can be found at approximately 50 to 70 mm depth. Blood flowing towards the probe will be originating from the MCA from this point, whereas blood flowing away from the probe will be flowing through the ACA. A headband held the probe in position so insonation depth and angle were maintained throughout the procedure (DWL Diamon Probe Fixation System; Compumedics DWL GmbH Singen, Germany).

*P*CO_2_ from exhaled air was obtained (Normocap 200; Datex-Ohmeda, Helsinki, Finland) via side-stream sampling. End-tidal partial pressure of CO_2_ (*P*_ET_CO_2_) was displayed during the measurements.

### Quantification of dynamic cerebral autoregulation

A description of the method to calculate dynamic CA in anaesthetised patients was published earlier by our group.^23^ In short, we used transfer function analysis, the most widespread method to quantify dynamic CA.^24^ CA is considered a linear, time-invariant high-pass filter, in which higher-frequency MAP oscillations (>0.15 Hz) are transferred passively to the cerebral circulation while lower-frequency (<0.15 Hz) MAP oscillations are dampened and delayed.^25^ Using transfer function analysis,^26^ dynamic CA, expressed as degrees (°), can be quantified as the phase lead between input (MAP) and output (MCA*V*_mean_) signals for a given frequency. In general, the magnitude of the phase lead is related to CA function. Earlier studies have suggested that a phase lead of 50° is consistent with intact CA, 30° indicates impaired CA and values nearing zero degrees indicate nonfunctional CA.^23,25,27^ Similarly, transfer function gain (expressed as cm s^−1^ mmHg^−1^) was calculated and reflects dampening of MCA*V*_mean_ signal variance in relation to MAP signal variance for a given frequency. Power spectra for MCA*V*_mean_ and MAP were computed using discrete Fourier transform in order to quantify signal variance.

The covariance between input and output signals can be expressed as coherence. This is the portion of MCA*V*_mean_ variance that can be attributed to MAP variance.^28^ The higher the coherence (which can have a value between 0 and 1), the more covariance between MAP and MCA*V*_mean_ oscillations at the various frequencies. Coherence of >0.5 was considered significant.

In awake individuals, lower frequency dynamic CA can be assessed easily because resting sympathetic activity results in a rhythmic variation in vascular tone, which translates into MAP oscillations of ∼0.1 Hz (known as Mayer waves).^29^ Unfortunately, during anaesthesia, there is dramatic reduction of sympathetic drive, resulting in the loss of the low-frequency MAP oscillations.^27,30^ High-frequency oscillations remain because of mechanical positive pressure ventilation.^31^

In this study, we applied our recently introduced method for mimicking Mayer waves during anaesthesia by adjusting the breathing frequency of the positive pressure mechanical ventilation to 6 breaths min^−1^ (=0.1 Hz).^28^

### Data collection and handling

Throughout surgery, two minute tracings of beat-to-beat MAP and MCA*V*_mean_ were recorded and visually inspected for artefacts. Segments containing visual artefacts were discarded. The remaining beat-to-beat data were interpolated (spline interpolation) and resampled to 4 Hz. To quantify the variability of MAP and MCA*V*_mean_, the power spectra of the two variables were estimated by transforming the time series of MAP and MCA*V*_mean_ with discrete Fourier transformation (DFT) to the frequency domain. From the cross spectrum density, transfer function phase shift and gain were derived.

Power was expressed as the averaged integrated area for the LF (low-frequency) range (0.07 to 0.15 Hz). The gain, as the ratio of the amplitudes of MCA*V*_mean_ and MAP, is taken to reflect the effective amplitude dampening of ABP fluctuations. To examine the strength of the relationship between MAP and MCA*V*_mean_, coherence was used to signify that the two cardiovascular signals co-vary significantly in the LF area. As with a correlation coefficient, it varies between 0 and 1, and a coherence above 0.5 was considered to provide a reliable estimate of the transfer function variables.

Phase shift was defined positive where MCA*V*_mean_ leads MAP. In healthy subjects, MCA*V*_mean_ leads MAP with 40 to 50° in the LF range.^23,27^ To account for the inter-subject variability, the gain was normalised for MAP and MCA*V*_mean_, and is expressed as the percentage change in cm s^−1^ per percentage change in mmHg.

### The FFT window

The MAP and MCA*V*_mean_ sequences of *N* points are divided into *K* sections of *M* points (where *M* is a power of two). Using an *M*-point FFT, successive segments are Hanning windowed with 50% overlapping samples. The full two minutes of beat-beat-beat data is used and the data is detrended and appended with zeroes to get a power of two length.

### Patient data collection

Information regarding patient characteristics and the surgical procedure, such as medical history, medication, and intra-operative haemodynamic parameters during surgery was collected from the electronic medical records.

### Study outcomes

The primary outcome of this study was response of dynamic CA during step-wise increases in MAP during propofol- and sevoflurane-based anaesthesia.

### Study protocol

Standard anaesthesia monitoring and study instrumentation were established upon arrival in the operating theatre. Anaesthesia was induced with sufentanil or remifentanil, lidocaine and propofol. The airway was maintained with either a laryngeal mask or tracheal intubation after muscle relaxation with rocuronium. Anaesthesia was maintained with either propofol (6 to 7 mg kg^−1^ h^−1^) or sevoflurane at target end tidal concentrations of < 1.2 minimum alveolar concentration (MAC) adjusted to age and remained unchanged during the measurements. The decision to use either intravenous or inhalational anaesthetics was made by the attending anaesthesiologist and not part of the study protocol. MAP was increased step-wise by use of increasing doses of intravenous phenylephrine infusion. Ventilator settings were according to standard clinical practice while a steady-state MAP was achieved. After reaching the target steady-state MAP (60, 70, 80, 90 and 100 mmHg), ventilation frequency was decreased to 6 min^−1^ to create 0.1 Hz oscillations in MAP. Tidal volume was increased to maintain minute ventilation. Furthermore, tidal volume was adjusted during the different steady-state blood pressure levels to keep the *P*_ET_CO_2_ stable. F_I_O_2_ was set to 0.4 and maintained during the measurements. PEEP was allowed to vary between 0 and 5 cmH_2_O. At least 3 min of data were recorded at each steady-state.

### Statistical analyses

Continuous data are presented as median with interquartile range [IQR], or as a mean ± SD when applicable. Normality of distribution was assessed visually using histograms and *Q*–*Q* plots, and statistically using the Shapiro–Wilkinson normality test. Differences between continuous data were analysed using Student's *t*-test or using the Wilcoxon rank-sum test when nonnormally distributed. Categorical data are presented as frequencies with percentages. Differences between categorical data were analysed using the Fisher's exact test. For each of the analyses *P* < 0.05 was considered statistically significant.

To test whether increasing MAP improves dynamic CA, we used a stepwise constructed linear mixed effect model. A base-model of CA in different MAP stages was constructed, with fixed intercept (mean). A random intercept, fixed slope and random slope were consecutively added in additional models. These nested models were compared using the −2 restricted log-likelihood (−2RLL) goodness-of-fit test. The model with the smallest number of parameters explaining most variance was selected. Both anaesthetic techniques were added to this model as a grouping factor, to analyse the base difference in CA with increasing blood pressure, between anaesthetic techniques. Finally, the relationship between increasing MAP and anaesthetic technique on change in CA was analysed by adding this interaction to the model. Other relevant transfer function variables, as well as haemodynamic, respiratory, and intra-operative medication variables were then added to the model in order to analyse potential systematic differences between anaesthetic techniques. Statistical analyses were performed using R, version 3.5.1,^32^ using the package ‘pwr’.^33^

### Sample size calculation

Other studies found phase lead varying between 30° to 52°, with SD varying between 5° and 10°.^23,27^ In this study, a 20% change in phase lead was considered clinically relevant. Based on the previous studies, we estimated the average phase lead at 40 ± 10°. Fifty-two patients would provide 80% power to detect an effect size of at least 0.8 at an estimated difference of 8°, with a 0.05 two-sided significance level. Anticipating an exclusion rate of 20%, minimum sample size was calculated at 66 patients.

## Results

### Patients

**Fig. 1:**
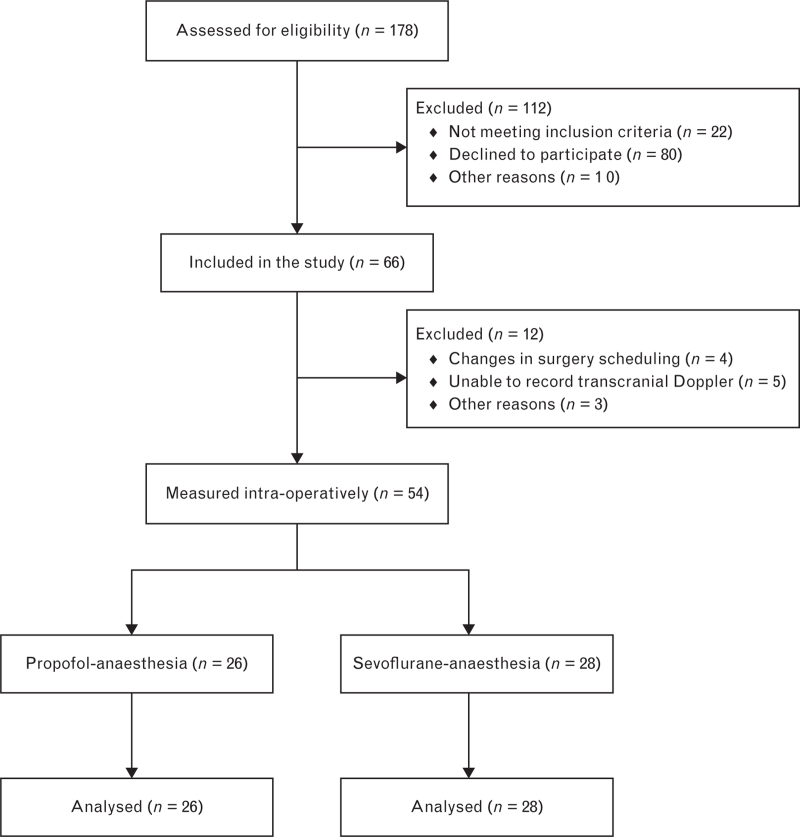
Study enrolment.

A total of 178 patients were screened for eligibility. Sixty-six patients were included from 11 January to 23 September 2019. Five patients had no suitable transcranial window to monitor MCA*V*_mean_, four patients could not follow study procedure due to last-minute changes in surgery scheduling after initial inclusion, one patient changed from ASA-II to ASA-III after inclusion, one patient experienced significant haemodynamic instability warranting deviation from protocol, and in one patient surgery was unexpectedly shortened, leaving not enough time to follow through on the study protocol. Further analysis of data was performed on the remaining 54 patients. Of those, 26 received propofol-based anaesthesia, and 28 received sevoflurane-based anaesthesia (Table 1). In general, there were no significant differences in baseline characteristics between both groups except for synthetic opioid regimen, with remifentanil being overrepresented compared to sufentanil in the propofol group (*P* = 0.001) (Fig. 1).

**Table 1:** Baseline characteristics

	Propofol	Sevoflurane
	*n* = 26	*n* = 28
Male, *n* (%)	18 (69.2)	17 (60.7)
Age (median [*Q*1*, Q*3])	49 [34, 59]	48 [37, 63]
Height (cm)	178 ± 8	178 ± 11
Weight (cm)	87 ± 18	89 ± 24
ASA classification, *n* (%)		
I	14 (53.8)	17 (60.7)
II	12 (46.2)	11 (39.3)
Systolic blood pressure (mmHg)	131 ± 15	128 ± 18
Diastolic blood pressure (mmHg)	78 ± 9	79 ± 12
Heart rate	73 ± 10	72 ± 11
Medication, *n* (%)		
Beta blockers	1 (3.8)	2 (7.1)
RAAS inhibitors	2 (7.7)	6 (21.4)
Diureticum	2 (7.7)	1 (3.6)
Ca antagonist	1 (3.8)	1 (3.6)
Statine	4 (15.4)	4 (14.3)
Anticoagulation	3 (11.5)	4 (14.3)
Relevant medical history, *n* (*%*)		
Coronary artery disease	1 (3.8)	0 (0.0)
Peripheral vascular disease	2 (7.7)	1 (3.6)
Other cardiac disease	1 (3.8)	1 (3.6)
Opioid, *n* (%)		
Sufentanil	16 (61.5)^∗^	28 (100.0)
Remifentanil	10 (38.5)^∗^	0 (0.0)
Surgery type, *n* (%)		
Gastro-enteral	0 (0.0)	1 (3.6)
Gynaecological	1 (3.8)	1 (3.6)
Orthopedical	3 (11.5)	6 (21.4)
Plastic	5 (19.2)	6 (21.4)
Traumatological	8 (30.8)	8 (28.6)
Urological	9 (34.6)	6 (21.4)

Values are presented as percentage (%), as median with lower and upper quartile [Q1 to Q3], or as mean ± SD. RAAS, renin–angiotensin–aldosterone system. ^∗^indicates *P* = 0.001 versus sevoflurane.

### Phenylephrine, respiratory and haemodynamic parameters

Phenylephrine was used to step-wise increase MAP. In patients anaesthetised with sevoflurane, more phenylephrine was used to achieve the desired MAP levels than when propofol was used (*P* = 0.001) (Table 2). By adjusting the tidal volume during the different steady state blood pressure levels, the P_ET_CO_2_ remained stable. Changes in tidal volume and peak inspiratory pressure (*P*_ip_) did not differ significantly between treatment groups at each steady-state MAP level (Table 3). F_I_O_2_ and PEEP remained unchanged throughout the procedure. S_P_O_2_ remained stable throughout the procedure. Changes in haemodynamic parameters between both groups throughout the different steady-state MAP levels did not differ systematically. Changes in CVRi differed significantly between propofol and sevoflurane regimens (*P* < 0.01) (Table 2).

**Table 2:** Descriptive statistics of haemodynamic parameters and phenylephrine infusion doses

Propofol (*n* = 26)						
MAP (mmHg)	Phenylephrine^∗^ (μg kg^−1^ h^−1^)	*P*_ET_CO_2_ (mmHg)	Heart rate (min^−1^)	Stroke volume (ml)	SVR (dyn s cm^−5^)	CVRi^∗^ (mmHg (cm s^−1^)^−1^)
63 ± 3	2 ± 4	41 ± 4	63 ± 14	77 ± 17	1257 ± 392	1.9 ± 0.5
72 ± 2	3 ± 4	41 ± 3	59 ± 13	85 ± 14	1199 ± 333	2.2 ± 0.6
80 ± 2	9 ± 7	42 ± 4	58 ± 11	91 ± 16	1246 ± 338	2.4 ± 0.6
90 ± 2	15 ± 10	41 ± 4	57 ± 9	93 ± 17	1443 ± 367	2.6 ± 0.7
100 ± 3	19 ± 15	41 ± 3	56 ± 10	99 ± 16	1615 ± 455	2.8 ± 0.8

Sevoflurane (*n* = 28)						
MAP (mmHg)	Phenylephrine^∗^ (μg kg^−1^ h^−1^)	*P*_ET_CO_2_ (mmHg)	Heart rate (min^−1^)	Stroke volume (ml)	SVR (dyn s cm^−5^)	CVRi^∗^ (mmHg (cm s^−1^)^−1^)
61 ± 4	3 ± 4	39 ± 4	58 ± 10	71 ± 13	1239 ± 277	1.5 ± 0.4
71 ± 2	11 ± 9	40 ± 4	59 ± 11	78 ± 13	1326 ± 328	1.6 ± 0.6
80 ± 2	19 ± 14	40 ± 4	57 ± 8	87 ± 17	1424 ± 293	1.7 ± 0.7
89 ± 2	26 ± 21	40 ± 2	58 ± 8	87 ± 17	1541 ± 367	1.9 ± 0.8
98 ± 4	25 ± 13	41 ± 2	56 ± 8	86 ± 18	1691 ± 332	2.0 ± 0.8

Mean arterial pressure (MAP) levels, partial end-tidal carbon dioxide pressure (*P*_ET_CO_2_), heart rate, stroke volume, systemic vascular resistance (SVR) and cerebrovascular resistance index (CVRi) during propofol- and sevoflurane-based anaesthesia at different steady-state blood pressure levels. Values are presented as mean ± SD. ^∗^ indicates phenylephrine infusion doses and CVRi differed systematically between propofol and sevoflurane anaesthesia (*P* < 0.01).

**Table 3:** Descriptive statistics of respiratory parameters

Propofol (*n* = 26)			Sevoflurane (*n* = 28)		
MAP (mmHg)	Tidal volume (ml)	*P*_IP_ (cmH_2_O)	MAP (mmHg)	Tidal volume (ml)	*P*_IP_ (cmH_2_O)
63 ± 3	697 ± 118	16 ± 4	61 ± 4	727 ± 80	16 ± 4
72 ± 2	755 ± 75	16 ± 3	71 ± 2	733 ± 108	17 ± 4
80 ± 2	763 ± 69	16 ± 3	80 ± 2	763 ± 155	18 ± 4
90 ± 2	707 ± 76	18 ± 3	89 ± 2	699 ± 100	19 ± 4
100 ± 3	810 ± 132	19 ± 3	98 ± 4	698 ± 68	22 ± 4

Mean arterial pressure (MAP), tidal volume and peak inspiratory pressure (*P*_ip_) during propofol- and sevoflurane-based anaesthesia when the ventilator frequency was set at 6 min^−1^. Values are mean ± SD.

### Dynamic cerebral autoregulation in relation to mean arterial pressure

During all MAP levels coherence was well above 0.5. Gain had a tendency to decrease as MAP increased in both groups (see Table 4).

**Table 4:** Descriptive statistics of transfer function analysis during paced breathing at 6 min^−1^ (0.1 Hz)

Propofol (*n* = 26)							
MAP (mmHg)	MCA*V*_mean_^∗^ (cm s^−1^)	MAP power (mmHg^2^ Hz^−1^)	MCA*V*_mean_ power (cm s^−1^)^2^ Hz^−1^	Coherence (k)	Phase lead^∗^ (°)	Gain (cm s^−1^) mmHg^−1^	Normalised gain (%/%)
63 ± 3	37 ± 15	3.8 ± 2.6	2.8 ± 3.2	0.87 ± 0.05	50 ± 20	0.68 ± 0.28	1.23 ± 0.62
72 ± 2	36 ± 12	5.2 ± 5.9	3.0 ± 4.0	0.82 ± 0.09	46 ± 21	0.69 ± 0.54	1.41 ± 0.90
80 ± 2	36 ± 12	5.1 ± 3.6	1.9 ± 1.6	0.78 ± 0.10	56 ± 21	0.65 ± 0.31	1.55 ± 0.78
90 ± 2	39 ± 13	5.4 ± 4.5	2.2 ± 2.5	0.77 ± 0.11	59 ± 20	0.60 ± 0.27	1.49 ± 0.76
100 ± 3	37 ± 12	6.2 ± 6.4	1.9 ± 1.7	0.78 ± 0.11	67 ± 23	0.54 ± 0.26	1.48 ± 0.93

Sevoflurane (*n* = 28)							
MAP (mmHg)	MCA*V*_mean_^∗^ (cm s^−1^)	MAP power (mmHg^2^ Hz^−1^)	MC*V*_mean_ power (cm s^−1^)^2^ Hz^−1^	Coherence (k)	Phase lead^∗^ (°)	Gain (cm s^−1^) mmHg^−1^	Normalised gain (%/%)
61 ± 4	44 ± 12	3.8 ± 3.0	2.3 ± 2.9	0.89 ± 0.07	44 ± 17	0.69 ± 0.27	0.96 ± 0.29
71 ± 2	50 ± 16	4.3 ± 3.0	2.3 ± 2.3	0.90 ± 0.09	46 ± 23	0.68 ± 0.25	0.96 ± 0.22
80 ± 2	53 ± 17	4.9 ± 3.9	1.8 ± 1.6	0.85 ± 0.08	47 ± 27	0.61 ± 0.26	0.94 ± 0.33
89 ± 2	54 ± 18	4.9 ± 3.8	2.3 ± 3.4	0.86 ± 0.07	49 ± 17	0.60 ± 0.26	0.92 ± 0.31
98 ± 4	55 ± 19	5.0 ± 4.5	1.4 ± 1.1	0.85 ± 0.08	56 ± 33	0.54 ± 0.36	0.98 ± 0.39

Mean arterial pressure (MAP), cerebral blood flow velocity (MCA*V*) and the amplitude of the oscillations in the MAP (MAP power) and the mean MCA*V* (MCA*V*_mean_ power) signal induced with a ventilation frequency of 6 min^−1^, coherence between the MAP and MCA*V*_mean_ oscillations, the phase lead of the MCA*V*_mean_ oscillation on the MAP oscillation and the MCA*V*_mean_ to MAP gain at different blood pressure levels in patients anaesthetised with propofol or sevoflurane. Expressed as mean ± SD. ^∗^ indicates MCA*V*_mean_ and phase lead differed systematically between propofol and sevoflurane anaesthesia (*P* < 0.01).

**Fig. 2:**
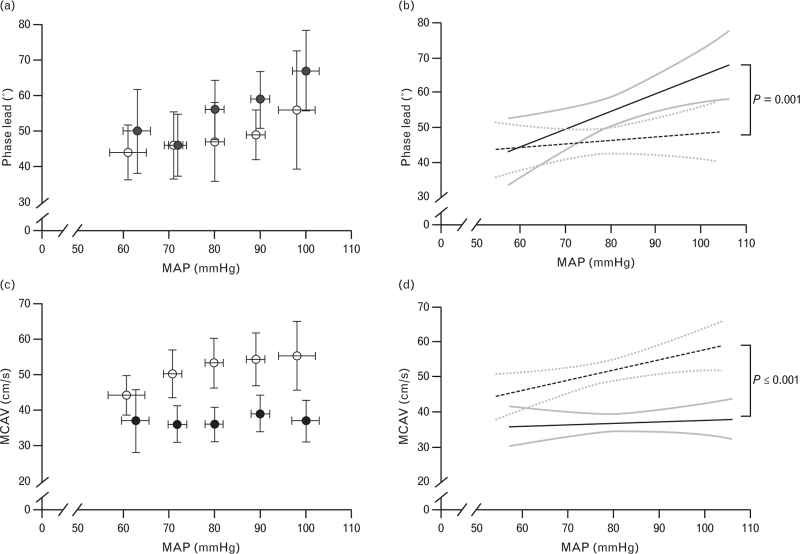
Relationship between MAP and dynamic CA expressed as low frequency MCA*V*_mean_-to-MAP phase-lead (°), and the relationship between MAP and MCA*V*_mean_. Closed circles/solid lines, propofol; open circle/dashed lines, sevoflurane. (a) Mean and standard error at every steady state MAP. (b) The linear regression with 95% confidence bands for dynamic CA. An increase in MAP was significantly associated with an increase in phase-lead in both the propofol-group (*P *≤* *0.001) and the sevoflurane-group (*P* = 0.04). In propofol-anesthetised patients, dynamic CA showed a higher increase per increasing unit of MAP, when compared to sevoflurane-anaesthetised patients (*P* = 0.001). (c) Mean and standard error at every steady state MAP. (d) The linear regression with 95% confidence bands for MCA*V*_mean_. An increase in MAP was significantly associated with an increase in MCA*V*_mean_ in both the propofol-group (*P *≤* *0.001) and the sevoflurane-group (*P *≤* *0.001). In sevoflurane-anaesthetised patients, MCA*V*_mean_ showed a higher increase per increasing unit of MAP, when compared to propofol-anaesthetised patients (*P *≤* *0.001). CA, cerebral autoregulation; MAP, mean arterial pressure; middle cerebral artery blood velocity (MCAV_mean_).

An increase in MAP was associated with an increase in phase-lead in both the propofol-group (0.73° mmHg^−1^, 95% CI 0.51 to 0.95, *P* ≤ 0.001) and the sevoflurane-group (0.21°·mmHg^−1^, 95% CI 0.01 to 0.42, *P* = 0.04). Furthermore, the anaesthetic technique showed an interaction with this association. In propofol patients, phase lead showed a 0.51° mmHg^−1^ higher increase per increasing unit of MAP, when compared to sevoflurane-anaesthetised patients (95% CI 0.207 to 0.820, *P* = 0.001) (Fig. 2a and b). The difference in phenylephrine between these groups had no modifying effect on the difference in increase in phase lead (*P* = 0.88).

### MCAV_mean_ in relation to mean arterial pressure

An increase in MAP was associated with an increase in MCA*V*_mean_ in both the propofol-group, 0.17 cm s^−1^ mmHg^−1^ (95%CI 0.08 to 0.26, *P*≤0.001) and the sevoflurane-group, 0.38 cm s^−1^ mmHg^−1^ (95%CI = 0.30 to 0.45, *P*≤0.001). Furthermore, the anaesthetic technique showed an interaction with this association. In sevoflurane patients, MCA*V*_mean_ showed a 0.21 cm s^−1^ higher increase per increasing unit of MAP, when compared to propofol-anaesthetised patients (95%CI 0.088 to 0.327, *P*≤0.001) (Table 4, Fig. 2c and d).

## Discussion

We found that dynamic CA efficacy improved when blood pressure was raised within the autoregulatory range. This observation was more pronounced during propofol than sevoflurane-based anaesthesia. We believe that the difference in vascular tone between sevoflurane and propofol could explain our observations and we will discuss this below.

### Static cerebral autoregulation

Traditional CA as proposed by Lassen is generally referred to as ‘static’ CA, and contains a plateau which has probably a slight positive slope.^34^ Volatile anaesthetics like sevoflurane are known to exacerbate this slope, while propofol does not.^8,11^ This difference is probably mediated by a difference in cerebrovascular tone.

Previous studies reported that during propofol-based anaesthesia, CBF did not change during phenylephrine,^35^ prostaglandin E1^36^ or nitroglycerine-induced modifications in blood pressure.^37^ Contrastingly, MCA*V*_mean_ increased together with blood pressure after phenylephrine and norepinephrine infusions in awake volunteers and during volatile-based anaesthesia.^8,38^ Our study generally confirms these findings, although we have found that MCA*V*_mean_ does increase slightly during propofol-based anaesthesia as MAP is being raised. This small increase should not be surprising, as we^39^ and others^34,40^ stated that a perfectly flat plateau of the CA curve is unlikely, because of the requirement of an infinite feedback gain, not generally observed in biological systems. However, it appears that static autoregulation is more effectively preserved during propofol-based anaesthesia than during sevoflurane-based anaesthesia.^8,11^

### Dynamic cerebral autoregulation

Dynamic CA has been found to be preserved during normal doses of sevoflurane,^27^ whereas it seems to be attenuated using higher doses of sevoflurane.^8^ On the other hand, dynamic autoregulation efficacy did not change during low- or high-dose propofol.^8,9,11,41^ In other situations of cerebrovascular dilation, such as after sodium nitroprusside^42^ or nitroglycerine infusion^43^ but also hypercapnia^7,44^ and high-dose isoflurane^8^ dynamic CA was impaired. Conversely, dynamic CA seemed improved during hypocapnia.^5,7^

### Implications for the anaesthesiologist

A unique feature of the present study is that it compared the effects of propofol and sevoflurane on both static as well as dynamic CA. The observations corroborate existing (circumstantial) evidence that propofol leads to cerebrovascular vasoconstriction and improved CA, while sevoflurane induces cerebrovascular vasodilation and impaired CA. Against the backdrop of this other evidence, a plausible explanation for these observations could be the different effects on cerebrovascular tone with both agents.

The results of this study further elucidate fundamental differences between propofol and volatile anaesthesia on cerebral haemodynamics. Importantly, there are two main implications. Firstly, while it has been recognised that there is a difference in cerebral vasomotor tone between anaesthetics, the behaviour of (dynamic) CA appears to depend on this very same difference. As far as we are aware, this relationship is rarely explicitly suggested. Secondly, the dogmatic view that the lower limit of CA is the inflection point under which cerebral hypoperfusion starts to occur is challenged. In fact, this is in accordance with earlier work,^45–47^ and the question remains whether the lower limit of CA has any relevance at all for anaesthesiologists.^3^ The results of this study support the view that CA is not an on-off mechanism and suggest that the lower limit is somewhat arbitrary, as dynamic CA continues to improve as MAP increases in the autoregulatory range.

### Limitations

Firstly, patients in our study were not randomised for anaesthetic regimen (specifically propofol versus sevoflurane). Rather, the agent for maintaining general anaesthesia was chosen by the attending anaesthesiologist. This could in theory have confounded our results, for which we attempted to control by statistical testing of differences in baseline patient characteristics.

Secondly, in our study we chose to increase MAP with phenylephrine. Although cerebral conductance vessels, like the middle cerebral artery, have α- and β-adrenergic innervation,^48^ their influence remains a matter of debate.^49^ In vivo, topical application of phenylephrine directly to dog pial artery segments lead to contraction.^50^ However, when injected directly into the internal carotid artery of dogs during cardiopulmonary bypass, there was no effect on CBF, presumably because phenylephrine does not cross the blood-brain barrier.^51^ There is no data in awake subjects clarifying the effect of phenylephrine infusion on cerebrovascular resistance and global CBF. When awake, if noninvasive cerebral blood flow measurements are used, phenylephrine infusion increases middle cerebral artery blood velocity ^52^ and leads to a NIRS-derived decrease in frontal lobe cerebral oxygenation^53,54^ while it attenuates dynamic CA.^18^

Thirdly, other potential cerebral vasomotor medication was given during surgery according to standard care. Most importantly, the use of remifentanil was overrepresented in the propofol-group in comparison to sufentanil, which may have contributed to the differences in intra-operative cerebral haemodynamic responses. One study found that in cardiac patients during remifentanil anaesthesia, moderate-dosed remifentanil had no effect on MCA*V*_mean_ while high-dose remifentanil decreased MCA*V*_mean_ by 31%.^55^ Others showed that remifentanil decreased MCA*V*_mean_ by ∼26% during anaesthesia, but showed no difference between low or high dosage.^56^ In addition, a study found no difference in MCA*V*_mean_ between children receiving remifentanil and propofol anaesthesia compared to children only given propofol.^57^ Furthermore, CA is preserved during propofol-remfentanil anaesthesia in humans.^58^ Sufentanil seems to cause similar effects on cerebral haemodynamics. One study in dogs under isoflurane and nitrous oxide anaesthesia showed a decrease in MCA*V*_mean_ of ∼35% after administration of normal-dose sufentanil.^59^ In humans with traumatic brain lesions and intracranial hypertension, MCA*V*_mean_ remained stable during normal-dose sufentanil as long as MAP remained unchanged. However, when MAP decreased, intracranial pressure (ICP) increased while MCA*V*_mean_ remained the same, suggesting that CA is maintained despite sufentanil infusion in this patient population.^60^ In summary, both sufentanil and remifentanil appear to have similar effects on CBF and CA during anaesthesia. Therefore, we do not expect that the choice of intra-operative opioids had a large effect on the current results.

Fourthly, the individual lower and upper limits of CA were not measured in this study. Therefore, these findings cannot be extrapolated to the complete range of the classical CA-curve. This is especially significant for the lower limits of CA (<50 mmHg), as deep hypotension is a common complication during surgery and is associated with ischaemic organ damage, such as in kidneys, myocardium and the central nervous system.^1,61,62^ However, studying this would require intentional induction of deep hypotension in humans, which is likely to raise serious ethical concerns.

Fifthly, intracranial pressure (ICP) was not measured in this study. Strictly speaking, CA only describes the pressure−flow relationship between cerebral perfusion pressure (CPP) and CBF. If ICP is increased, there is a possibility of measuring ‘false autoregulation’.^63^ During such a situation, CPP may remain normal if ICP increases equally to MAP. However, whereas resistance changes are mediated by changing intracranial vascular calibre in the healthy brain, resistance changes may be mediated by for example brain oedema or haemorrhage in the diseased brain. So, pressure autoregulation in this sense is not a physiological mechanism but rather pathophysiological, and may confound results found in specific patient populations, especially those with increased ICP as a result of trauma. The chosen population for this study was relatively healthy with no active intracranial disease. As far as we know, no patients demonstrated clinical evidence of increased ICP. Furthermore, intrathoracic pressure always remained below 30 cmH_2_O (see Table 3), making venous jugular congestion more unlikely.

Sixthly, in an attempt to limit the use of vasopressors and vasodilators and thus prevent large swings in MAP, we decided not to randomise the MAP levels. The consequence might be that any possible confounding effect due to medication rather than the MAP itself cannot be ruled out (Table 2).

Seventhly, transfer function analysis as used here does not take directional sensitivity of dynamic CA into account. Earlier research has suggested the existence of such mechanism,^64^ although there has been debate whether this is a true phenomenon or a by-product of experimental interventions.^65^

Finally, measurement of CBF using TCD is indirect because it measures the blood velocity in the MCA MCA*V*_mean_ estimates CBF under the assumption that MCA diameter does not change and that flow in the centre of the vessel is laminar.^66^ Indeed, MCA diameter hardly changed despite fluctuations in MAP or CO_2_ during direct observation during craniotomy in 12 patients.^67^ However, most of these patients were undergoing surgery after subarachnoid haemorrhage. Recent studies in healthy subjects using MRI to measure the diameter of the proximal MCA segments suggest that modest vessel calibre changes occur during hypocapnia and hypercapnia.^68,69^ One study showed that MCA diameter changes during rhythmic hand grip exercises.^70^ It is unclear whether these changes were due to blood pressure fluctuations, or increased sympathetic activity, of which the latter is virtually absent during anaesthesia. Studies have shown that changes in MCA*V*_mean_ accurately reflect changes in CBF.^71,72^ Therefore, MCA*V*_mean_ has been a widely accepted method of estimating changes in CBF. However, because the posterior circulation was not measured, the current findings cannot be generalised beyond the anterior circulation.

## Conclusion

We confirmed that there is a gradient in the autoregulatory range during propofol and is even more pronounced, during sevoflurane-based anaesthesia. Dynamic CA improves as blood pressure increases (using phenylephrine) during propofol- and sevoflurane-based anaesthesia. This effect is more pronounced for propofol than for sevoflurane. We hypothesise that cerebrovascular tone may be an important underlying mechanism.
